# Modulating *OCA2* Expression as a Promising Approach to Enhance Skin Brightness and Reduce Dark Spots

**DOI:** 10.3390/biom14101284

**Published:** 2024-10-11

**Authors:** Eunbyul Cho, Kyong Eun Hyung, Yun-Ho Choi, Hyeyeon Chun, Daehyun Kim, Seung-Hyun Jun, Nae-Gyu Kang

**Affiliations:** LG Household and Health Care, R & D Center, Seoul 07795, Republic of Korea; goodstar@lghnh.com (E.C.); hke0512@lghnh.com (K.E.H.); youknow@lghnh.com (Y.-H.C.); chunhy@lghnh.com (H.C.); daehyun@lghnh.com (D.K.)

**Keywords:** pigmentation, skin tone, dark spots, melanogenesis, cosmetic ingredient, oculocutaneous albinism II (*OCA2*)

## Abstract

The oculocutaneous albinism II (*OCA2*) gene encodes a melanosomal transmembrane protein involved in melanogenesis. Recent genome-wide association studies have identified several single nucleotide polymorphisms within *OCA2* genes that are involved in skin pigmentation. Nevertheless, there have been no attempts to modulate this gene to improve skin discoloration. Accordingly, our aim was to identify compounds that can reduce *OCA2* expression and to develop a formula that can improve skin brightness and reduce hyperpigmented spots. In this study, we investigated the effects of *OCA2* expression reduction on melanin levels, melanosome pH, and autophagy induction through siRNA knockdown. Additionally, we identified several bioactives that effectively reduce *OCA2* expression. Ultimately, in a clinical trial, we demonstrated that topical application of those compounds significantly improved skin tone and dark spots compared to vitamin C, a typical brightening agent. These findings demonstrate that *OCA2* is a promising target for the development of efficacious cosmetics and therapeutics designed to treat hyperpigmentation.

## 1. Introduction

Skin pigmentation is a visually distinguishing characteristic that is crucial in perceptions of beauty and attractiveness across cultures and societies [1]. For instance, Asian cultures traditionally favor light skin and a flawless complexion as symbols of beauty [2]. Furthermore, as individuals age, their skin tends to become duller and acquire more dark spots. So, lighter and more even skin tones are often associated with a more youthful appearance [3]. These preferences have created a constant demand for methods to achieve a desired skin color and uniformity, such as whitening cosmetics, makeup products, and dermatological procedures to ameliorate pigmentation.

Understanding the biological mechanism underlying skin pigmentation is essential to develop these methods effectively. Skin color is determined by the melanin pigment produced by melanocytes [4]. The type, production, and distribution of melanin are influenced by the complex interplay between genetic, environmental, and evolutionary factors. Among these, genetic factors play a significant role in determining the skin pigmentation range in different populations [5]. Recent advancements in the field of genomics have enabled researchers to identify specific genes associated with variations in skin color through genome-wide association studies (GWASs).

Previous GWASs have demonstrated that various single nucleotide polymorphisms (SNPs) within the *OCA2* gene are strongly associated with skin pigmentation. For instance, rs74653330 and rs1800414 have been identified as key determinants of skin color in Asians [6]. Particularly in Korean populations, rs74653330 has shown the strongest correlation with the *L (skin brightness) and *b (skin yellowness) parameters of the CIELAB indices, commonly used to measure skin tone [7]. Other SNPs, such as rs11855019 and rs12913832 have been linked to an increased prevalence of freckles [8,9]. The strong associations between multiple SNPs in *OCA2* and skin pigmentation suggest it as a potential target for developing treatments to improve skin tone and reduce dark spots—the main motivation of the present study.

At the cellular level, the *OCA2* gene encodes the “p protein”, an integral melanosomal membrane protein essential for melanin production [10,11]. This protein is implicated in melanosome neutralization, a process integral to melanosome maturation [12,13]. Melanosomal pH directly influences tyrosinase activity, the key enzyme in melanin synthesis [14,15]. While OCA2 functions as a chloride channel, its precise role in pH regulation remains unclear [16]. Previous studies indirectly suggested *OCA2* KD leads to melanosome acidification, but direct measurement was lacking. This study aims to directly assess OCA2’s impact on melanosomal pH and further elucidate its associated factors.

Despite the well-established role of OCA2 in regulating skin pigmentation across multiple levels and its potential as a skin-brightening target, no research to date has focused on modulating *OCA2* expression to achieve this goal. In this study, we aimed to address this gap by identifying *OCA2* expression down-regulatory substances and evaluating their clinical application for skin depigmentation purposes. Furthermore, we investigated the potential role of OCA2 in autophagy, a cellular process known to degrade melanosomes to gain a deeper understanding of its function in melanogenesis [17,18,19]. We demonstrated the superior efficacy of *OCA2*-down-regulating substances compared to vitamin C, a well-known skin-brightening agent, in improving skin tone and reducing dark spots. Our results suggest that *OCA2* is a promising target for future cosmetics and therapeutics with aims related to pigmentation.

## 2. Materials and Methods

### 2.1. Cell Culture

The mouse melanoma B16F10 cell line (CRL-6475) was obtained from the American Type Culture Collection (ATCC) (Manassas, VA, USA). The B16F10 cells were maintained in Dulbecco’s Modified Eagle’s medium (Gibco, Grand Island, NY, USA) with the addition of 10% fetal bovine serum (Gibco), 400 U/mL penicillin (Gibco), and 50 g/mL streptomycin (Gibco). The cells were kept between passage numbers 4 and 12. They were incubated at 37 °C in an atmosphere of 5% CO_2_.

### 2.2. Transfection of B16F10 with siRNA

B16F10 cells were plated in six-well plates and cultured for 24 h to ensure complete attachment, reaching 70–80% confluence. The cells were then transiently transfected with 30 pmole scrambled siRNA and *OCA2*-specific siRNA, respectively (Bioneer, Daejeon, Republic of Korea) using Lipofectamine RNAiMax (Invitrogen, Carlsbad, CA, USA) following the manufacturer’s instructions. Four hours post-transfection, the reaction mixture was removed, and the culture medium was replaced with the fresh complete media.

### 2.3. RT-qPCR

B16F10 cells were cultured at 60% confluency. After 24 h, when cells were well adherent, they were subjected to active ingredients treatment or siRNA transfection. The active ingredients were treated at various concentrations for 24 h and the siRNAs were transfected as described above in Section 2.2. The media were then changed to complete media containing various concentrations of bioactive molecules.

After the ingredients were treated for 24 h, RNA was extracted using the AccuPrep Universal RNA Extraction Kit (Bioneer). The concentration and purity of the extracted RNA were measured using a NanoDrop spectrophotometer (ThermoFisher Scientific, Waltham, MA, USA). An amount of 1 μg of total RNA was reverse transcribed into cDNA using a cDNA synthesis kit (Bioneer) according to the manufacturer’s instructions, utilizing a Veriti 96-Well Thermal Cycler (Applied Biosystems, Foster City, CA, USA). The cDNA amplification was carried out with the TaqMan Universal PCR Master Mix (Applied Biosystems) according to the manufacturer’s instructions, utilizing the StepOnePlusTM RT-PCR system (Applied Biosystems). The PCR protocol involved 40 cycles with the following conditions: denaturation at 95 °C for 45 s, annealing at 60 °C for 1 min, and extension at 72 °C for 45 s. qPCR was conducted using commercially available TaqMan primers (ThermoFisher Scientific): *GAPDH* for mouse (Mm99999915_g1), *OCA2* for mouse (Mm00498969_m1) and for human (Hs00609330_m1), and *SLC45A2* for mouse (Mm00499728_m1). Additionally, *GAPDH* for human was amplified using GAPDH OLIGO MIX (Applied Biosystems).

### 2.4. Measurement of Melanin Contents

B16F10 cells were seeded into six-well plates and incubated for 24 h. Afterward, they were either treated with 10 nM of alpha-melanocyte-stimulating hormone (α-MSH) or left untreated. The cells were then washed twice with phosphate-buffered saline (PBS) and collected. Cell lysis was performed by incubating in 100 μL of 1 N NaOH at 80 °C for 30 min. The lysates were transferred to a 96-well plate, and the absorbance was measured at 405 nm. Subsequently, the total protein content of the cell lysates was determined using the Pierce BCA Protein Assay kit (ThermoFisher Scientific). The melanin content, measured at 405 nm, was normalized to the total protein content.

### 2.5. Measurement of Cellular Tyrosinase Activity

Tyrosinase activity assay was conducted in 96-well plates by adding 50 µL of cell extract and 10 µL of 1 mM L-DOPA in 0.1 M phosphate buffer (pH 7.2). After incubating for 1 h at 37 °C, the absorbance was recorded at 475 nm. The values were divided by the total protein content for normalization.

### 2.6. Western Blotting

B16F10 cells were seeded in 60 mm dishes and transfected with siRNA as described above. Subsequently, the cells were treated with bafilomycin A1 (Sigma-Aldrich, St. Louis, MO, USA, 5.08409) for 24 h. After treatments, cells were washed with ice-cold phosphate-buffered saline and lysed on ice in M-PER buffer (ThermoFisher Scientific) supplemented with cOmplete^TM^ protease inhibitor cocktail and phosphatase inhibitor (Roche, Basel, Switzerland). A total of 35 μg of protein was analyzed by Western blotting using appropriate antibodies to assess protein expression. The resulting blot was quantitated by chemiluminescence detector iBright FL1500 (ThermoFisher Scientific).

The following antibodies were used for antibody incubation: anti-LC3B (ab192890) and anti-beta actin (ab8227) from Abcam (Cambridge, UK), anti-rabbit IgG, HRP-linked Antibody (7074s) from Cell Signaling Technology (Danvers, MA, USA), and anti-OCA2 (PA5-102825) from ThermoFisher Scientific. The concentration of the antibody depends on the instructions by the manufacturer.

### 2.7. Fluorescence Microscopy for Analyzing Acidity of Melanosome and Vesicles

Cells were seeded and incubated for two days following treatment with bioactive compounds and siRNA transfection. For lysotracker staining, the media were aspirated and cells were incubated with 100 nM LysoTracker^TM^ Red DND-99 (Invitrogen, L7528) in serum-free DMEM for 1 h at 37 °C. The cells were then washed three times with PBS, then fixed with 4% paraformaldehyde. To quantify the acidified vacuoles, the nuclei were stained with 1 µg/mL 4′,6-diamidino-2-phenylindole (DAPI) for 10 min.

To assess the acidification of melanosomes, Tyrosinase-related protein 1 (TYRP1) and DAPI were further co-stained. Cells were permeabilized with 0.1% Triton X-100 in PBS and blocked with 3% BSA in PBST for 1 h. Next, cells were incubated with TYRP1 antibody (Abcam, AB109012) overnight at 4 °C, washed with PBS, and further incubated with anti-rabbit secondary antibody Alexa Fluor™ 488 (Invitrogen, A-11034) at a 1:1000 dilution for 30 min. Cells were again washed in PBS three times and stained with DAPI. Fluorescence images were captured using an EVOS FL Auto 2 imaging system.

Vesicle acidity was evaluated by counting the lysotracker-stained puncta, which was then normalized to the total cell number. The size and intensity of acidic lysosomes were analyzed using the “Analyze Particles” function in ImageJ software.

The acidified melanosomes were analyzed by defining regions of interest (ROIs) for TYRP1 and Lysotracker. The “threshold” function of ImageJ was employed to ascertain the areas of TYRP1 and Lysotracker. The common areas of the two images were identified through the “image multiply” function. The number and size of the regions were analyzed using the “Analyze Particles” function. The number of puncta was normalized by dividing the number of puncta by the amount of DAPI.

### 2.8. LC3B Puncta Analysis to Detect Autophagy Induction

B16F10 cells were treated with the ingredients for 24 h. Then, cells were fixed with 4% paraformaldehyde for 10 min and washed with PBS. Cells were permeabilized with 0.1% Triton-X-100 in PBS for 10 min and blocked with 3% BSA in PBST for 1 h. For LC3B staining, the cells were incubated with LC3B primary antibodies (1:1000 in 1% BSA in PBST; Abcam, ab192890) overnight at 4 °C, washed with PBST three times, and treated with anti-rabbit secondary antibody (1:1000 in dilution buffer; A11034; Thermo Fisher Scientific, Carlsbad, CA, USA) for 30 min at room temperature. Cell images were obtained using an EVOS FL Auto2 imaging system. The number of LC3B cells were analyzed using the “Analyze Particles” function in the ImageJ software (version 1.54f).

### 2.9. Human Clinical Trial

This study received approval from the ethics committee of the LG H&H Institutional Review Board (LGHH-SKEV-2022-09-A). Healthy Korean volunteers participated in the clinical trial. Pregnant women and individuals undergoing skin treatments were excluded. The trial was conducted in a double-blind format.

For the skin tone assessment, a total of 11 Korean individuals participated in the study, including 4 females and 7 males, with a mean age of 37.9 years (standard deviation [SD] = 6.62). In contrast, the dark spot assessment focused on individuals presenting with visible dark spots of at least 1 mm in diameter on their faces. A total of 12 participants were enrolled in this assessment, including 8 females and 4 males, with a mean age of 35.72 years (SD = 6.73).

The test formulations, Lumi-OCA2 and the 8% vitamin C formulation, shared the same inactive ingredients. Lumi-OCA2 contained the following active ingredients: 0.007% genistein, 0.003% quercetin, 0.1% polydatin, and 1% zinc pyrrolidone (Zinc PCA). The 8% vitamin C formulation served as the positive control and contained 8% ascorbic acid as its active ingredient. Both formulations were applied following the same schedule: topically to the face twice daily, every morning and evening, for four weeks. The specific application areas may vary depending on the evaluation method. Before measurements, participants rested for at least 20 min in a controlled environment (humidity: 45 ± 5%, temperature: 22 ± 2 °C) after facial cleansing.

For skin tone measurement, a half-face blinding method was used with the control formulation applied to the left side of the face and Lumi-OCA2 to the right side. Skin tone was measured in triplicate on both cheeks using a chromameter (CM-700d; Konica Minolta, Tokyo, Japan) at the start of the trial and after four weeks to evaluate changes in skin pigmentation.

For the pigmentation score assessment, participants with dark spots were included. Among the 12 individuals, 6 used the vitamin C formula, and the remaining 6 applied Lumi-OCA2 to their dark spots. Dark spots were measured in triplicate using a topographic skin measurement device (Antera 3D CS; Miravex, Dublin, Ireland) at the beginning of the trial and after four weeks to analyze the pigmentation score.

### 2.10. Statistical Analysis

The data are presented as mean values with standard error of the mean (SEM) derived from at least three independent experiments. Statistical significance was evaluated using Student’s *t*-test, with a significance level of * *p* < 0.05, ** *p* < 0.01, and *** *p* < 0.001. To ensure the appropriate application of Student’s *t*-test, we assessed the normality of the data using the Shapiro–Wilk test in GraphPad Prism (version 8). The results confirmed that the data met the normality assumption (*p* > 0.05).

## 3. Results

### 3.1. Reduction in Melanin Synthesis Due to OCA2 Knockdown (KD)

To investigate the role of the *OCA2* gene in melanogenesis, we employed an siRNA system to modulate *OCA2* expression. B16F10 cells were transfected with either scramble siRNA (siScram) as a negative control or *OCA2*-specific siRNA (siOCA2). To assess the impact of *OCA2* knockdown (KD) under conditions that promote melanogenesis, we also treated the cells with α-MSH.

**Figure 1:**
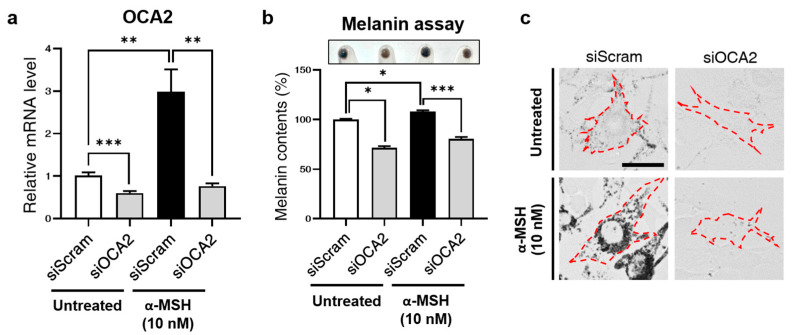
Decreased melanin production due to *OCA2* KD in B16F10. (**a**) Alteration in mouse *OCA2* expression following *OCA2* KD in α-MSH-treated conditions compared to untreated controls (n = 3, with duplicates for each group). (**b**) Change in melanin contents following *OCA2* KD in control (untreated) compared to α-MSH-treated conditions (n = 4 for each group). (**c**) Representative images of melanin production changes (brightfield). Scale bar = 37 μm. Red dashed lines indicate cell boundaries. Error bars indicate the standard error of the mean. * *p* < 0.05, ** *p* < 0.01, *** *p* < 0.001; Student’s *t*-test.

First, we observed a reduction in *OCA2* mRNA levels upon transfection with siOCA2 (Figure 1a), which was further confirmed by a decrease in OCA2 protein levels in the siOCA2 groups as assessed by Western blot analysis (Supplementary Figure S1a,b). The siOCA2-transfected group exhibited significantly lower mRNA levels compared to the siScram-transfected group. Interestingly, while α-MSH treatment markedly increased *OCA2* mRNA levels in control cells, this increase was reversed in the *OCA2* KD group.

Subsequently, we assessed melanin content in *OCA2* KD cells to validate its role in melanogenesis. As shown in Figure 1b,c, melanin content decreased in the *OCA2* KD group, consistent with previous reports [20]. In line with the qPCR data, melanin levels increased upon α-MSH treatment but were subsequently reduced following *OCA2* KD.

### 3.2. Acidification of Melanosome Following OCA2 KD

**Figure 2:**
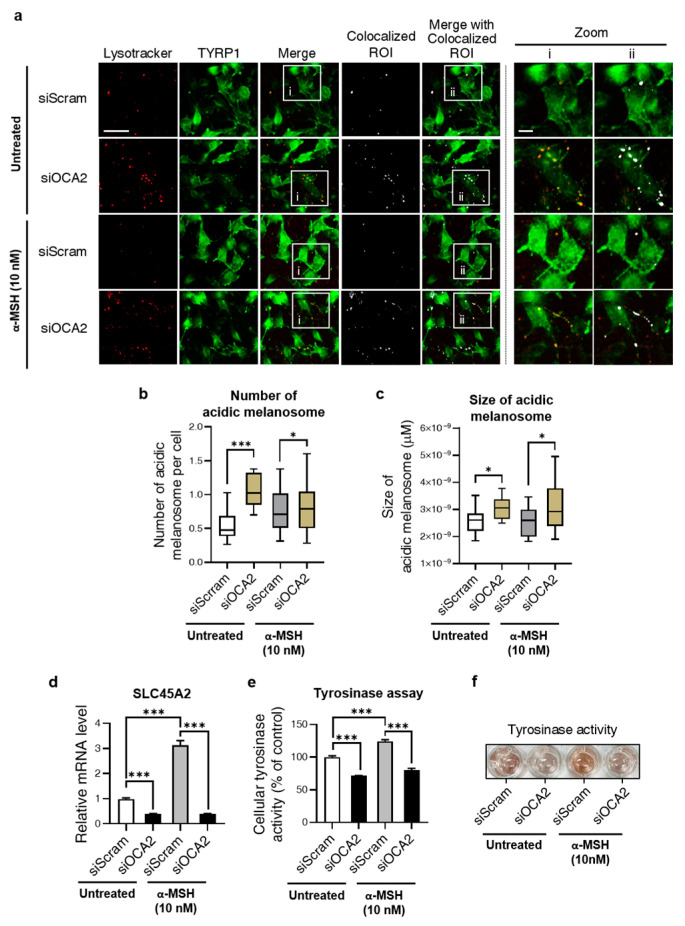
Acidification of the melanosomes and alterations in the molecules related to melanosomal pH regulation following *OCA2* KD. (**a**) Representative fluorescence images with colocalized ROI analysis of lysotracker (red) and TYRP1 (green) (siScram + untreated, n = 1662 cells; siOCA2 + untreated, n = 707 cells; siScram + α-MSH, n = 811 cells, siOCA2 + α-MSH, n = 1415 cells). Scale bar = 46 μm (zoomed image: 12.3 μm). (**b**,**c**) Quantification analysis of colocalized ROI puncta of Lysotracker and TYRP1 from the image in (**a**). Analysis of the number (**b**) and size (**c**) of colocalized puncta upon *OCA2* KD. (**d**) Change in expression of *SLC45A2* due to *OCA2* KD (n = 3, with duplicates for each group). (**e**) Quantification data of cellular tyrosinase activity following *OCA2* KD (n = 4 for each group). (**f**) Representative image of tyrosinase activity in tyrosinase assay. Error bars indicate the standard error of the mean. * *p* < 0.05, *** *p* < 0.001; Student’s *t*-test.

Given the role of OCA2 in neutralizing melanosomal pH, we investigated how its KD affects this process. Considering the opposite effect of neutralization, we analyzed melanosomal acidification following *OCA2* KD. For this purpose, cells were co-stained with Lysotracker, a marker of acidic vesicles, and the TYRP1 antibody to label melanosomes. As shown in Figure 2a, acidic melanosomes were identified by extracting their colocalized regions of interest (ROIs). For a comprehensive analysis, the number and mean size of the colocalized ROIs were measured following *OCA2* KD. As a result, Figure 2b,c show that both values significantly increased, confirming melanosomal acidification. This trend was consistent in α-MSH-treated groups.

Furthermore, we investigated other factors related to melanosome pH in the *OCA2* KD condition. These include (1) solute carrier family 45 member 2 (*SLC45A2*), a proton-associated glucose and sucrose transporter known to contribute to maintaining neutral pH during melanosome maturation, and (2) tyrosinase, whose activity and degradation are known to be determined by melanosome pH [12,14,15,21].

When assessing the expression level of *SLC45A2*, we observed a significant reduction upon *OCA2* KD, as shown in Figure 2d. Furthermore, the increase in mRNA levels under α-MSH treatment, and their reversal upon *OCA2* KD, exhibited a similar pattern of change with *OCA2* expression. In both conditions, *OCA2* KD significantly suppressed *SLC45A2* expression levels, as displayed in Figure 2d. Similar effects were also observed for tyrosinase activity: as shown in Figure 2e,f, tyrosinase activity was reduced following *OCA2* KD. These observations indicate that *OCA2* KD acidifies melanosomes while concurrently influencing associated factors, including *SLC45A2* expression and tyrosinase activity. This process ultimately results in the inhibition of melanin production.

### 3.3. Role of OCA2 in Regulating Autophagy

Previous studies demonstrated that *OCA2* KD diminishes the bafilomycin A1-induced increase in melanin production [22,23]. Bafilomycin A1, a lysosome pH neutralizer, also inhibits autophagy flux [17,18]. Autophagy induction is known to degrade melanosomes, thereby inhibiting melanin synthesis. Therefore, we hypothesized that autophagy induction contributes to the decreased melanin observed in *OCA2* KD cells.

**Figure 3:**
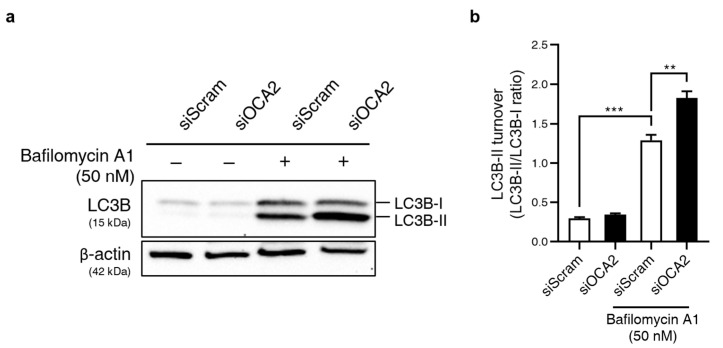
*OCA2* KD regulates autophagy. (**a**) Western blot analysis of LC3B protein expression upon *OCA2* KD in B16F10 cells with or without bafilomycin A1 treatment (50 nM). β-actin was used as a loading control. Original images can be found in Supplementary Materials. (**b**) Quantification of LC3B-II turnover, calculated as the ratio of LC3B-II expression to LC3B-I (n = 4 for each group). Error bars indicate the standard error of the mean. ** *p* < 0.01, *** *p* < 0.001; Student’s *t*-test.

Autophagy induction was analyzed by assessing the conversion of LC3B-I to LC3B-II (measured as the LC3B-II/LC3B-I ratio) using immunoblotting [24]. As shown in Figure 3a,b, the LC3B-II/LC3B-I ratio was significantly increased upon bafilomycin A1 treatment. This indicates an accumulation of autophagosomes due to inhibition of the latter stage of autophagy. This ratio, already elevated by bafilomycin A1 treatment, was further increased significantly in *OCA2* KD cells. These results suggest that *OCA2* KD induces autophagosome formation. Therefore, we propose that autophagy induction is likely involved in the decrease in melanin production observed upon *OCA2* KD.

### 3.4. Identification of OCA2-Modulating Substances for Potential Skin-Brightening Effects

**Figure 4:**
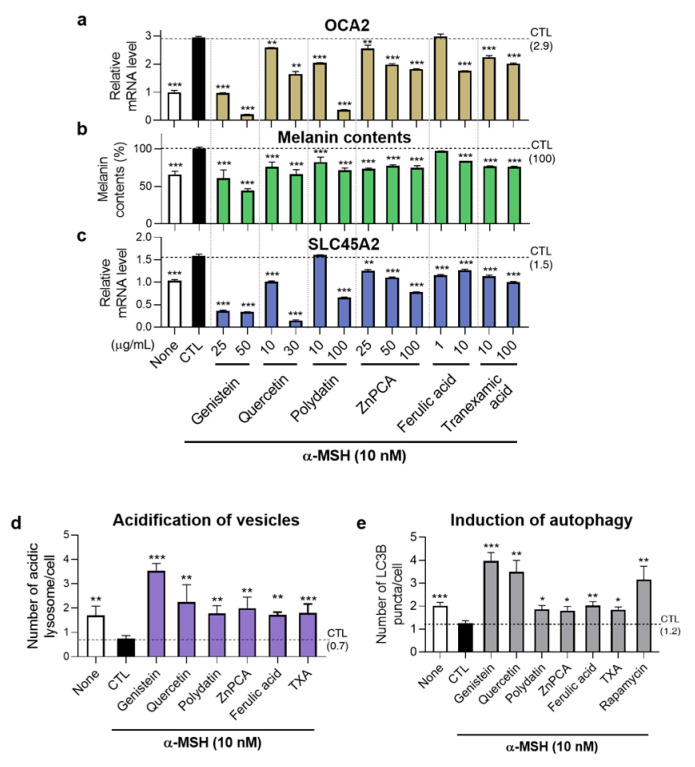
Effects of *OCA2* expression-regulating substances in melanin production, expression of *SLC45A2*, melanosome acidification, and autophagy induction. (**a**) Identification of *OCA2* expression down-regulating material in B16F10 cells (n = 3, with duplicates for each group). (**b**,**c**) Decrease in melanin production (**b**) and alteration of *SLC45A2* expression (**c**) following treatment with the six *OCA2* expression-regulating substances (n = 4 for each groups in (**b**); n = 6 for each groups in (**c**)). (None: negative control; CTL: control; TXA: tranexamic acid). (**d**) Quantitative graph of number of acidic vesicular organelles in B16F10 cells through acridine orange staining by treating *OCA2*-down-regulating substances (n = 332, 529, 228, 174, 381, 419, 683, 317 for each group). (Genistein: 25 μg/mL, Quercetin: 10 μg/mL, Polydatin: 100 μg/mL, ZnPCA: 25 μg/mL, Ferulic acid: 10 μg/mL, TXA: 10 μg/mL). (**e**) Quantitative graph of autophagy induction by analyzing LC3B puncta number in B16F10 cells treated with the six substances. The concentration of substances is same with (**d**) (n = 329, 706, 193, 172, 675, 380, 422, 293, 367 for each group). Error bars indicate the standard error of the mean. * *p* < 0.05, ** *p* < 0.01, *** *p* < 0.001; Student’s *t*-test.

After confirming the active role of OCA2 in regulating melanosome pH, its associated factors, autophagy, and ultimately melanin production, we screened a library of substances to identify compounds that can modulate *OCA2* expression. We discovered a total of fifteen bioactives that actively suppress *OCA2* expression. The effects of six of these are shown in Figure 4, while the effects of the remaining nine are summarized in Supplementary Figure S3.

As shown in Figure 4a, treating B16F10 cells with genistein, quercetin, polydatin, ZnPCA, ferulic acid, and tranexamic acid (TXA) for one day significantly reduced *OCA2* expression, confirming their efficacy as *OCA2*-modulating substances. We also investigated whether these compounds had a similar effect on *OCA2* expression in human primary melanocytes. Treatment with four of these substances (genistein, quercetin, polydatin, and ZnPCA) significantly reduced human *OCA2* expression in Normal Human Epithelial Melanocyte (HEMn-LP) cells (Supplementary Figure S4a).

Consequently, similar to observations in *OCA2* KD cells, decreased *SLC45A2* expression and acidification of vesicular organelles containing melanosomes were observed under treatment with these bioactives (Figure 4c–e, Supplementary Figure S5a,b). Specifically, vesicular acidification was measured using LysoTracker staining, which showed an overlap of approximately 60% to 90% with the melanosome marker TYRP1 (Supplementary Figure S6). Most importantly, these bioactives also suppressed melanin production in B16F10 cells (Figure 4b). These results highlight the potential of these bioactives as melanin-regulating substances, showing promise for cosmetic and therapeutic applications.

We now discuss the underlying mechanism of how our identified bioactives regulate *OCA2* expression (Supplementary Figure S7). We note that some of these *OCA2* down-regulating compounds—genistein, quercetin, ferulic acid, epigallrocatechine gallate (EGCG), and luteolin—belong to the polyphenolic phytochemical classes. These polyphenolic phytochemicals not only act as antioxidants but also are known to inhibit mitochondrial ATPase activity [25].

We thus aimed to understand whether mitochondrial ATPase activity plays a major role in regulating *OCA2* expression. To investigate this, we treated B16F10 cells with oligomycin, a mitochondrial ATPase inhibitor. As shown in Supplementary Figure S7a, oligomycin treatment decreased *OCA2* expression in a dose-dependent manner, supporting a correlation between *OCA2* expression and mitochondrial ATPase activity. This trend was also observed in HEMn-LP cells, primary human melanocytes (Supplementary Figure S4b). Furthermore, *SLC45A2* expression, which was reduced upon *OCA2* KD, exhibited a similar pattern to *OCA2* following oligomycin treatment (Supplementary Figure S8).

In contrast, treating B16F10 cells with N-acetylcysteine (NAC), an artificial antioxidant, did not affect the *OCA2* expression as shown Supplementary Figure S7b. Taken together, our results thus suggest that the inhibition of mitochondrial ATPase activity by polyphenolic bioactives we identified might be involved in the regulation of *OCA2* expression.

### 3.5. Clinical Evaluation of Pigmentation Reduction by Topical Application of OCA2 Down-Regulating Substance

Ultimately, we tested the effects of these *OCA2* down-regulating bioactives on depigmentation at a clinical level. To achieve this, we formulated a topical cream containing *OCA2*-regulating substances (genistein, quercetin, polydatin, and ZnPCA) and named it Lumi-OCA2. We compared the effect of Lumi-OCA2 on skin tone and pigmented spots with vitamin C (8%), a widely recognized depigmentation agent.

**Figure 5:**
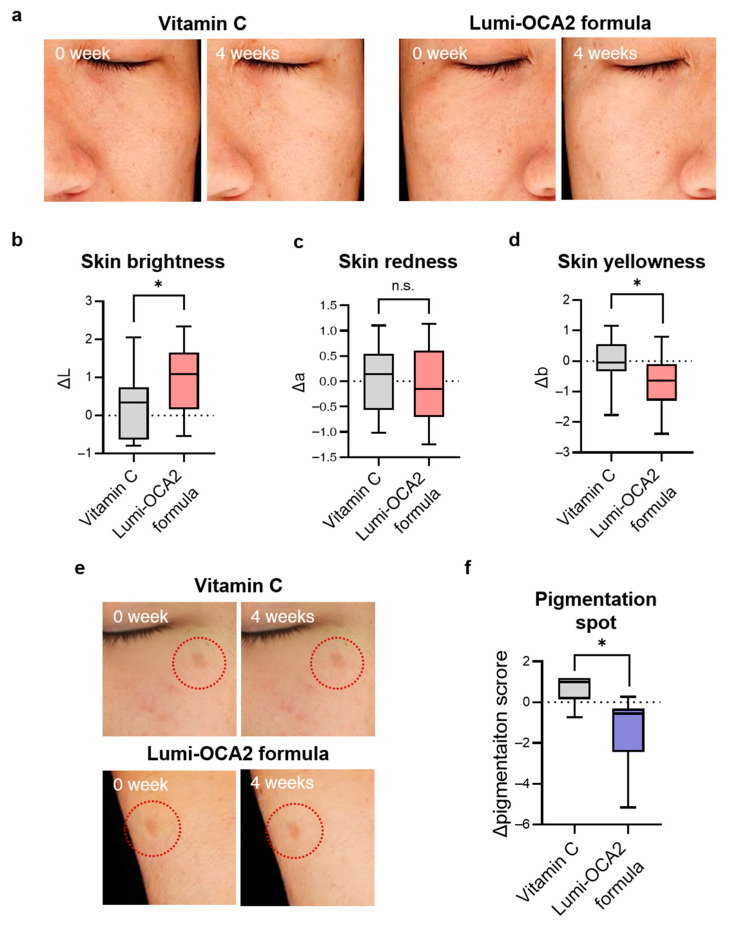
Clinical evaluation of pigmentation reduction by topical application of Lumi-OCA2 formula, which contains *OCA2*-regulating substances. (**a**) Representative images of skin tone improvement after 4 weeks of using Lumi-OCA2 formula compared to vitamin C (8%) formula (blind test, half and half test, n = 11). (**b**–**d**) Quantification of ΔL: skin lightness (**b**), Δa: skin redness (**c**), Δb: skin yellowness (**d**) change for measuring skin tone using a spectrophotometer. (**e**) Representative images demonstrating improvement in pigmentation spots before and after topical application of the Lumi-OCA2 formula. (**f**) Quantitative analysis of dark spot pigmentation score using a topographic skin measurement device, Antera 3D. Pigmentation score was significantly reduced when applying the Lumi-OCA2 formula compared to the vitamin C (8%) formula (blind test; a total of 12 participants, with n = 6 in both vitamin C and Lumi-OCA2 formula groups). Error bars indicate the standard error of the mean. n.s. (not significant); * *p* < 0.05; Student’s *t*-test.

First, skin tone improvement was unbiasedly evaluated by a half-and-half test. Participants applied the Lumi-OCA2 formula on one side of the face and the vitamin C (8%) formula on the other side. After four weeks, the skin tone on both sides was measured using a chromameter, and the changes in the value *L (skin lightness), *a (skin redness), and *b (skin yellowness) were analyzed. As shown in Figure 5b, the application of the Lumi-OCA2 formula improved skin lightness by more than three times compared to vitamin C. Skin yellowness was also better improved with the Lumi-OCA2 formula (Figure 5d). In contrast, the change in skin redness was limited (Figure 5c). Our findings on the manner in which *OCA2* downregulation affects skin tone-related values are consistent with previous studies. It was reported that the SNP (rs74653330) in *OCA2* is hypomorphic and associated with skin tone value and *L and *b values, but not *a values [20,26].

We further investigated the improvement in the degree of pigmentation in dark spots. The participants were divided into two groups, with half receiving the vitamin C formula and half receiving the Lumi-OCA2 formula. After applying the formulation to dark spots twice a day for four weeks, we measured and analyzed the pigmentation level using a topographic skin measurement device, Antera 3D. Figure 5f demonstrated that the Lumi-OCA2 formula had superior effects on dark spots compared to vitamin C.

## 4. Discussion

In summary, we identified and validated the clinical efficacy of materials that regulate *OCA2*, a gene associated with skin pigmentation. Additionally, we investigated several factors that could affect melanogenesis upon *OCA2* KD. For instance, *OCA2* KD induced melanosomal acidification and autophagy, accompanied by a decrease in the expression of *SLC45A2*, a melanosomal pH regulator.

Our multi-faceted investigation into the effects of *OCA2* KD revealed a notable increase in the mean size of acidic melanosome (Figure 2c). This observation is particularly noteworthy given that *OCA2*-deficient cells exhibit a markedly reduced presence of mature, stage IV melanosomes [12]. In light of this, our results in Figure 2b suggest that in *OCA2* KD cells, acidic melanosomes tend to accumulate at stage III rather than earlier stages (I/II). As melanin synthesis primarily occurs in these later stages, their acidification likely contributes significantly to the observed depigmentation. This underscores the critical role of OCA2 in both melanosome maturation and acidification, emphasizing the need for further research into how acidification at different melanogenesis stages influences melanin synthesis.

As shown in Figure 2d, *OCA2* KD decreased expression of *SLC45A2*, a melanosome pH regulator, but the underlying mechanism remains unclear. One possibility is that OCA2, either alone or through its interaction with SLC45A2, may act as a transcriptional regulator of *SLC45A2*. However, this possibility seems less likely given the previous findings that OCA2 is localized to melanosomes, lysosomes, and the endoplasmic reticulum, rather than the nucleus [12,27,28]. Alternatively, the functional relationship between OCA2 and SLC45A2 may lead to negative autoregulation of *SLC45A2* expression. Indeed, David L. Duffy et al. (2010) reported a significant epistatic interaction between alleles of these two genes, and Linh Lea et al. (2013) observed that SLC45A2 compensates for the reduced melanin synthesis caused by *OCA2* KD [12,29]. Thus, it is plausible that the reduction in OCA2 protein levels could impact SLC45A2 function (e.g., stability, localization, or activity), potentially due to their shared signaling pathway, ultimately affecting its own gene expression. However, further research is needed to elucidate this mechanism. While OCA2 is known to influence melanosome pH, it transports Cl^-^ and not H^+^ ions directly [16]. Therefore, our study proposes that OCA2 might participate in melanosome pH regulation through functional interaction with other H^+^ transporters, such as SLC45A2.

Unexpectedly, treatment with oligomycin, a mitochondrial ATPase inhibitor, significantly decreased *OCA2* expression (Supplementary Figure S7a). This change in gene expression, linked to mitochondrial dysfunction, aligns with the well-established concept of retrograde signaling between mitochondrial and nucleus [30,31]. The underlying mechanisms may involve alterations in mitochondrial proteostasis, ATP, ROS, and calcium production, which can influence the activity of transcription factors such as NRF, HIF-1α, and CREB [32]. Additionally, previous studies have reported changes in the expression of other genes following oligomycin treatment, supporting our hypothesis that *OCA2* expression is regulated by this mechanism [33,34]. This finding will provide valuable insights for the identification of new ingredients that regulate *OCA2* expression for therapeutic or cosmetic applications.

Topical application of *OCA2*-down-regulating substances led to a decrease in skin yellowness (b*), as shown in Figure 5d. Two potential mechanisms may explain this: (1) Pheomelanin reduction: While pheomelanin contributes to skin yellowness, OCA2 is primarily involved in eumelanin synthesis, not pheomelanin. This is supported by a mouse model with *OCA2* mutation showing reduced eumelanin but unchanged pheomelanin levels [35]. (2) Enhanced AGE degradation: AGEs, yellow-brown compounds accumulating in the epidermis, contribute to skin yellowness [36,37]. They are degraded via autophagy [37,38], a process we have shown to be induced by *OCA2* KD (Figure 3). This aligns with studies linking epidermal autophagy to changes in skin b* values [21]. This study suggests that autophagy induction by *OCA2* down-regulation may potentially contribute to modulating skin tone, particularly in reducing yellowness, paving the way for further research on improving skin dullness and achieving a healthier complexion.

The modulation of *OCA2* expression could offer a promising avenue for therapeutic intervention in pigmentation disorders. Genetic studies have linked *OCA2* polymorphisms and mutations to the pigmentation disorders ranging from solar lentigiens (rs4778138, rs1800414) to oculocutaneous albinism type II [39,40,41]. This highlights the promise of targeting *OCA2* for therapeutic intervention. Our identified bioactives, which regulate *OCA2* expression, demonstrate potential for treating hypermorphic *OCA2*-related disorders. At the same time, we recognize the need to explore strategies to enhance *OCA2* expression for treating hypopigmentation disorders associated with loss-of-function *OCA2* variants, including albinism. In conclusion, our research underscores the significant role of OCA2 in pigmentation and its potential as a therapeutic target for a spectrum of pigmentation disorders.

In this study, we gained clinical insights into the regulation of *OCA2* expression, a GWAS-identified gene associated skin pigmentation. Building upon this, we will expand our research to investigate the potential bioactives that regulate the expression of two other GWAS-identified genes (Supplementary Figure S9a,b). One such gene is RAB11 Family Interacting Protein 2 (*RAB11FIP*), associated with freckles, and the other is Tumor protein P63 (*TP63*), related to solar lentigines [42,43,44,45,46]. Unlike previous research focused on *MITF* and *TYR*, we emphasize the complex interplay of multiple genes in determining skin pigmentation [47,48,49]. We will further explore the mechanism of other pigmentation-related genes and identify novel ingredients regulating their expression. This will contribute to the development of highly personalized cosmetics and pharmaceuticals for skin discoloration.

## 5. Conclusions

This study focused on targeting *OCA2*, a GWAS-identified gene associated with skin pigmentation. We confirmed its potential for improving pigmentation clinically and identified novel bioactive compounds that down-regulate *OCA2*, elucidating their mechanisms of action. This approach can be applied to discover additional ingredients for modulating *OCA2* expression. Clinical trials demonstrated the superior skin-brightening effects of these compounds compared to existing agents, validating our strategy. This study expands the focus beyond traditional melanogenesis-related genes (e.g., *TYR*, *MITF*) to highlight the importance of targeting diverse genes associated with skin pigmentation. Our findings provide a framework for the development of cosmetics and therapeutics aimed at improving skin tone.

## Data Availability

The datasets generated and/or analyzed during the current study are available from the corresponding author(s) upon reasonable request.

## Supplementary Materials

The following supporting information can be downloaded at: https://www.mdpi.com/article/10.3390/biom14101284/s1, Figure S1: Reduced OCA2 protein level upon *OCA2* KD in B16F10 cells. Figure S2: Uncropped western blot images corresponding to Figure 3. Figure S3: Additionally identified *OCA2* expression-decreasing bioactives: Tannic acid, Gallic acid hydrate, Luteolin, Bakuchiol, Aspergillus ferment, Gamma oryzanol, Sunflower oil, Isostearic acid, and Oil soluble licorice. Figure S4: Alteration of human *OCA2* expression in HEMnLP cells upon treatment with *OCA2* regulators. Figure S5: *OCA2* regulator treatment induces vesicle acidification and autophagy. Figure S6: Colocalization analysis between Lysotracker and TYRP1 following *OCA2* KD in B16F10 cells. Figure S7: Reduction in *OCA2* expression through treatment with oligomycin, mitochondrial ATPase inhibitor in B16F10 cells. Figure S8: Reduction in *SLC45A2* mRNA level upon treatment with oligomycin in B16F10 cells. Figure S9: Identification of expression regulatory substances for *RAB11FIP2* and *TP63*. Table S1: RT-qPCR.

## References

[1] Dimitrov D., Kroumpouzos G. (2023). Beauty perception: A historical and contemporary review. Clin. Dermatol..

[2] Li E.P.H., Min H.J., Belk R.W., Kimura J., Bahl S. (2008). Skin lightening and beauty in four Asian cultures. Adv. Consum. Res..

[3] Fink B., Grammer K., Matts P.J. (2006). Visible skin color distribution plays a role in the perception of age, attractiveness, and health in female faces. Evol. Hum. Behav..

[4] Visscher M.O. (2017). Skin Color and Pigmentation in Ethnic Skin. Facial Plast. Surg. Clin. N. Am..

[5] Naik P.P., Farrukh S.N. (2022). Influence of Ethnicities and Skin Color Variations in Different Populations: A Review. Ski. Pharmacol. Physiol..

[6] Eaton K., Edwards M., Krithika S., Cook G., Norton H., Parra E.J. (2015). Association study confirms the role of two OCA2 polymorphisms in normal skin pigmentation variation in East Asian populations. Am. J. Hum. Biol..

[7] Seo J.Y., You S.W., Shin J.-G., Kim Y., Park S.G., Won H.-H., Kang N.G. (2022). GWAS Identifies Multiple Genetic Loci for Skin Color in Korean Women. J. Investig. Dermatol..

[8] Barón A.E., Asdigian N.L., Gonzalez V., Aalborg J., Terzian T., Stiegmann R.A., Torchia E.C., Berwick M., Dellavalle R.P., Morelli J.G. (2014). Interactions between ultraviolet light and MC1R and OCA2 variants are determinants of childhood nevus and freckle phenotypes. Cancer Epidemiol. Biomark. Prev..

[9] Duffy D.L., Montgomery G.W., Chen W., Zhao Z.Z., Le L., James M.R., Hayward N.K., Martin N.G., Sturm R.A. (2007). A three-single-nucleotide polymorphism haplotype in intron 1 of OCA2 explains most human eye-color variation. Am. J. Hum. Genet..

[10] Park S., Morya V.K., Nguyen D.H., Singh B.K., Lee H.-B., Kim E.-K. (2015). Unrevealing the role of P-protein on melanosome biology and structure, using siRNA-mediated down regulation of OCA2. Mol. Cell. Biochem..

[11] Oetting W.S., Garrett S.S., Brott M., King R.A. (2005). P gene mutations associated with oculocutaneous albinism type II (OCA2). Hum. Mutat..

[12] Le L., Escobar I.E., Ho T., Lefkovith A.J., Latteri E., Haltaufderhyde K.D., Dennis M.K., Plowright L., Sviderskaya E.V., Bennett D.C. (2020). SLC45A2 protein stability and regulation of melanosome pH determine melanocyte pigmentation. Mol. Biol. Cell.

[13] Peterson K.A., Neuffer S., Bean M.E., New L., Coffin A.B., Cooper C.D. (2019). Melanosome maturation proteins Oca2, Mitfa and Vps11 are differentially required for cisplatin resistance in zebrafish melanocytes. Exp. Dermatol..

[14] Ancans J., Tobin D.J., Hoogduijn M.J., Smit N.P., Wakamatsu K., Thody A.J. (2001). Melanosomal pH controls rate of melanogenesis, eumelanin/phaeomelanin ratio and melanosome maturation in melanocytes and melanoma cells. Exp. Cell Res..

[15] Zeng H., Harashima A., Kato K., Gu L., Motomura Y., Otsuka R., Maeda K. (2017). Degradation of tyrosinase by melanosomal pH change and a new mechanism of whitening with propylparaben. Cosmetics.

[16] Bellono N.W., Escobar I.E., Lefkovith A.J., Marks M.S., Oancea E. (2014). An intracellular anion channel critical for pigmentation. eLife.

[17] Murase D., Kusaka-Kikushima A., Hachiya A., Fullenkamp R., Stepp A., Imai A., Ueno M., Kawabata K., Takahashi Y., Hase T. (2020). Autophagy declines with premature skin aging resulting in dynamic alterations in skin pigmentation and epidermal differentiation. Int. J. Mol. Sci..

[18] Kovacs D., Cardinali G., Picardo M., Bastonini E. (2022). Shining Light on Autophagy in Skin Pigmentation and Pigmentary Disorders. Cells.

[19] Lee K.W., Kim M., Lee S.H., Kim K.D. (2022). The Function of Autophagy as a Regulator of Melanin Homeostasis. Cells.

[20] Sviderskaya E.V., Bennett D.C., Ho L., Bailin T., Lee S.-T., Spritz R.A. (1997). Complementation of hypopigmentation in p-mutant (pink-eyed dilution) mouse melanocytes by normal human P cDNA, and defective complementation by OCA2 mutant sequences. J. Investig. Dermatol..

[21] Bin B.-H., Bhin J., Yang S.H., Shin M., Nam Y.-J., Choi D.-H., Shin D.W., Lee A.-Y., Hwang D., Cho E.-G. (2015). Membrane-associated transporter protein (MATP) regulates melanosomal pH and influences tyrosinase activity. PLoS ONE.

[22] Manga P., Orlow S.J. (2001). Inverse Correlation Between Pink-Eyed Dilution Protein Expression and Induction of Melanogenesis by Bafilomycin A1. Pigment Cell Res..

[23] Chen K., Manga P., Orlow S.J. (2002). Pink-eyed Dilution Protein Controls the Processing of Tyrosinase. Mol. Biol. Cell.

[24] Klionsky D.J., Abdel-Aziz A.K., Abdelfatah S., Abdellatif M., Abdoli A., Abel S., Abeliovich H., Abildgaard M.H., Abudu Y.P., Acevedo-Arozena A. (2021). Guidelines for the use and interpretation of assays for monitoring autophagy (4th edition). Autophagy.

[25] Zheng J., Ramirez V.D. (2000). Inhibition of mitochondrial proton F0F1-ATPase/ATP synthase by polyphenolic phytochemicals. Br. J. Pharmacol..

[26] Yuasa I., Umetsu K., Harihara S., Miyoshi A., Saitou N., Park K.S., Dashnyam B., Jin F., Lucotte G., Chattopadhyay P.K. (2007). OCA2*481Thr, a hypofunctional allele in pigmentation, is characteristic of northeastern Asian populations. J. Hum. Genet..

[27] Bellono N.W., Escobar I.E., Oancea E. (2016). A melanosomal two-pore sodium channel regulates pigmentation. Sci. Rep..

[28] Sitaram A., Piccirillo R., Palmisano I., Harper D.C., Dell’Angelica E.C., Schiaffino M.V., Marks M.S. (2009). Localization to Mature Melanosomes by Virtue of Cytoplasmic Dileucine Motifs Is Required for Human OCA2 Function. Mol. Biol. Cell.

[29] Duffy D.L., Zhao Z.Z., Sturm R.A., Hayward N.K., Martin N.G., Montgomery G.W. (2010). Multiple pigmentation gene polymorphisms account for a substantial proportion of risk of cutaneous malignant melanoma. J. Investig. Dermatol..

[30] Kleine T., Leister D. (2016). Retrograde signaling: Organelles go networking. Biochim. Biophys. Acta-Bioenerg..

[31] Da Cunha F.M., Torelli N.Q., Kowaltowski A.J. (2015). Mitochondrial Retrograde Signaling: Triggers, Pathways, and Outcomes. Oxidative Med. Cell. Longev..

[32] Butow R.A., Avadhani N.G. (2004). Mitochondrial signaling: The retrograde response. Mol. Cell.

[33] Allen A.M., Graham A. (2012). Mitochondrial function is involved in regulation of cholesterol efflux to apolipoprotein (apo)A-I from murine RAW 264.7 macrophages. Lipids Health Dis..

[34] Lee Y.L., Chiao C.H., Hsu M.T. (2011). Transcription of muscle actin genes by a nuclear form of mitochondrial RNA polymerase. PLoS ONE.

[35] Hirobe T., Ito S., Wakamatsu K. (2011). The mouse pink-eyed dilution allele of the P-gene greatly inhibits eumelanin but not pheomelanin synthesis. Pigment. Cell Melanoma Res..

[36] Chen C.-Y., Zhang J.-Q., Li L., Guo M.-M., He Y.-F., Dong Y.-M., Meng H., Yi F. (2022). Advanced Glycation End Products in the Skin: Molecular Mechanisms, Methods of Measurement, and Inhibitory Pathways. Front. Med..

[37] Laughlin T., Tan Y., Jarrold B., Chen J., Li L., Fang B., Zhao W., Tamura M., Matsubara A., Deng G. (2020). Autophagy activators stimulate the removal of advanced glycation end products in human keratinocytes. J. Eur. Acad. Dermatol. Venereol..

[38] Ott C., Jacobs K., Haucke E., Santos A.N., Grune T., Simm A. (2014). Role of advanced glycation end products in cellular signaling. Redox Biol..

[39] Peng Q., Huels A., Zhang C., Yu Y., Qiu W., Cai X., Zhao Y., Schikowski T., Merches K., Liu Y. (2023). Genetic Variants in Telomerase Reverse Transcriptase Contribute to Solar Lentigines. J. Investig. Dermatol..

[40] Jiang B., Zhang H., Kan Y., Gao X., Du Z., Liu Q. (2024). Novel compound heterozygous mutations in *OCA2* gene were identified in a Chinese family with oculocutaneous albinism. Mol. Genet. Genom. Med..

[41] Fernandez L.P., Milne R.L., Pita G., Floristan U., Sendagorta E., Feito M., Avilés J.A., Martin-Gonzalez M., Lázaro P., Benítez J. (2009). Pigmentation-related genes and their implication in malignant melanoma susceptibility. Exp. Dermatol..

[42] Maillard A., Alby C., Gabison E., Doan S., Caux F., Bodemer C., Hadj-Rabia S. (2019). P63-related disorders: Dermatological characteristics in 22 patients. Exp. Dermatol..

[43] Liu L., Nielsen F.M., Emmersen J., Bath C., Hjortdal J., Riis S., Fink T., Pennisi C.P., Zachar V. (2018). Pigmentation Is Associated with Stemness Hierarchy of Progenitor Cells Within Cultured Limbal Epithelial Cells. Stem Cells.

[44] Endo C., Johnson T.A., Morino R., Nakazono K., Kamitsuji S., Akita M., Kawajiri M., Yamasaki T., Kami A., Hoshi Y. (2018). Genome-wide association study in Japanese females identifies fifteen novel skin-related trait associations. Sci. Rep..

[45] Wang P., Sun X., Miao Q., Mi H., Cao M., Zhao S., Wang Y., Shu Y., Li W., Xu H. (2022). Novel genetic associations with five aesthetic facial traits: A genome-wide association study in the Chinese population. Front. Genet..

[46] Watanabe T., Tahira M., Morino S., Horie T., Adachi K., Tsutsumi R., Yamada N., Yoshida Y., Yamamoto O. (2013). Novel morphological study of solar lentigines by immunohistochemical and electron microscopic evaluation. J. Dermatol..

[47] Qian W., Liu W., Zhu D., Cao Y., Tang A., Gong G., Su H. (2020). Natural skin-whitening compounds for the treatment of melanogenesis (Review). Exp. Ther. Med..

[48] Kim H.-E., Ishihara A., Lee S.-G. (2012). The effects of caffeoylserotonin on inhibition of melanogenesis through the downregulation of MITF via the reduction of intracellular cAMP and acceleration of ERK activation in B16 murine melanoma cells. BMB Rep..

[49] Draelos Z.D., Diaz I., Cohen A., Mao J., Boyd T. (2020). A novel skin brightening topical technology. J. Cosmet. Dermatol..

