# Supplementary material for: Modulating OCA2 Expression as a Promising Approach to Enhance Skin Brightness and Reduce Dark Spots
Source: Biomolecules. 2024 Oct 11;14(10):1284. doi: 10.3390/biom14101284 (PMC11506640; doi:10.3390/biom14101284)
Supplement: Supplementary file 1 [file biomolecules-14-01284-s001.zip › biomolecules-3209542-supplementary.pdf]

biomolecules

MDPI

Article

Modulating OCA2 expression as a promising approach to enhance skin brightness and reduce dark spots

Eunbyul Cho†, Kyong Eun Hyung†, Yun-Ho Choi, Hyeyeon Chun, Daehyun Kim, Seung-Hyun Jun* and Nae-Gyu Kang*

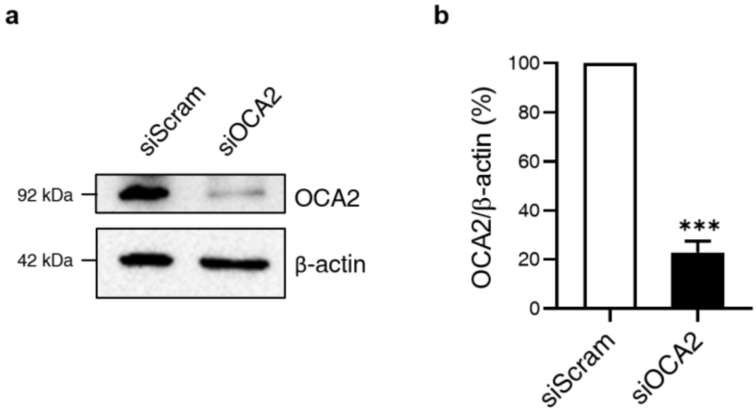

Supplementary Figure S1. Reduced OCA2 protein level upon OCA2 KD in B16F10 cells. **(a)** Western blot analysis following scramble siRNA (siScram) and OCA2 siRNA (siOCA2) transfection. β-actin was used as a loading control. **(b)** Quantification data of OCA2 protein expression normalized to B-actin. OCA2 expression upon siScram transfection was normalized to 100%. The protein expression of OCA2 was significantly decreased upon siOCA2 KD. *** $p < 0.001$; (n = 3 for each group; Student's t-test).

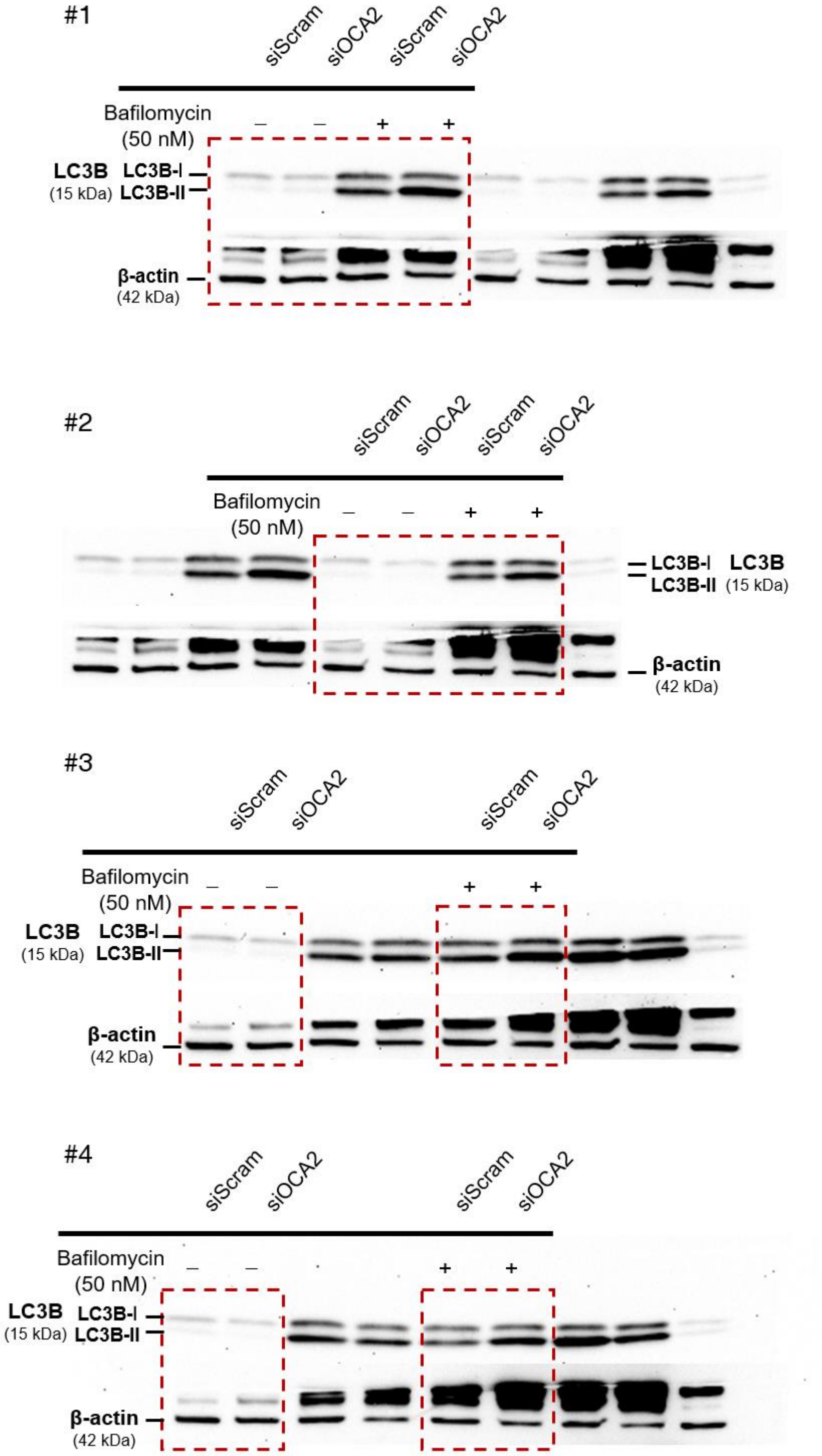

Supplementary Figure S2. Uncropped western blot images corresponding to Figure 3. OCA2 KD further enhances the Bafilomycin A1-induced increase in the LC3B-II/LC3B-I ratio in B16F10 cells. Western blots were performed four times independently.

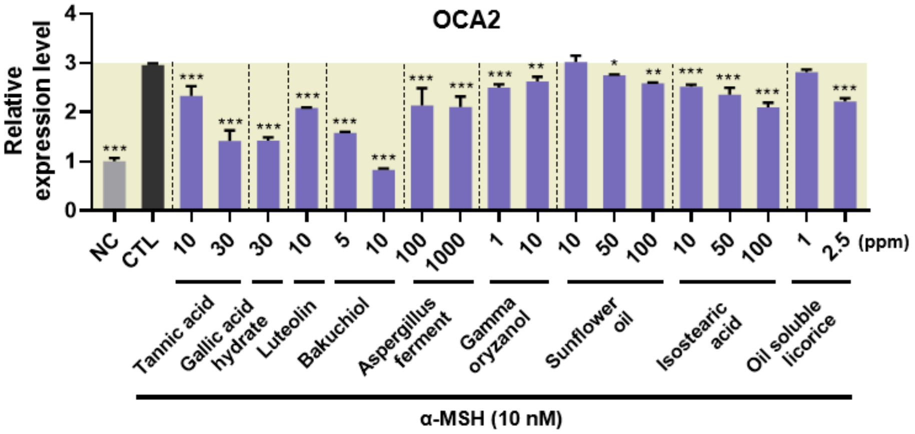

Supplementary Figure S3. Additionally identified OCA2 expression-decreasing bioactives: Tannic acid, Gallic acid hydrate, Luteolin, Bakuchiol, *Aspergillus* ferment, Gamma oryzanol, Sunflower oil, Isostearic acid and Oil soluble licorice. Error bars indicate the standard error of the mean. * $p < 0.05$, ** $p < 0.01$, *** $p < 0.001$; (n = 3 for each group; Student's *t*-test).

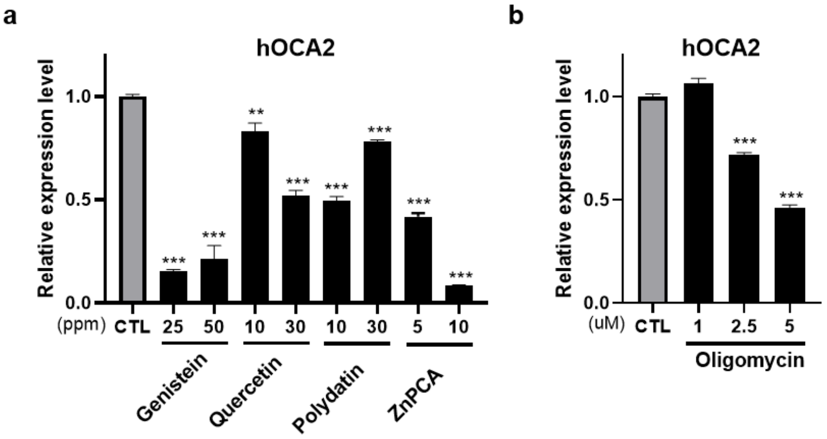

Supplementary Figure S4. Alteration of human OCA2 expression in HEMnLP cells upon treatment with OCA2 regulators. **(a)** The reduction of hOCA2 expression by genistein, quercetin, polydatin and ZnPCA treatment. (n = 3 for each group). **(b)** The concentration-dependent reduction of hOCA2 mRNA levels by the mitochondrial ATPase inhibitor oligomycin. Error bars indicate the standard error of the mean. ** $p < 0.01$, *** $p < 0.001$; (n = 3 for each group; Student's *t*-test).

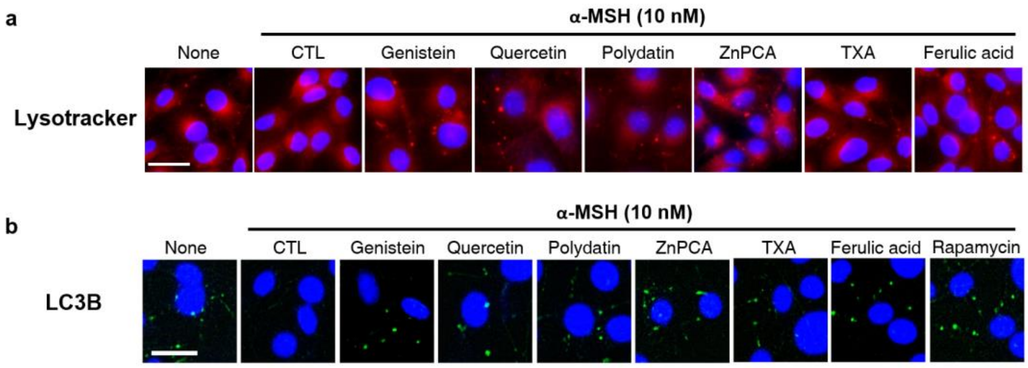

Supplementary Figure S5. OCA2 regulator treatment induces vesicle acidification and autophagy. **(a-b)** Representative images of **(a)** increased Lysotracker puncta (red) and **(b)** increased LC3B puncta (green) upon OCA2 regulator treatment. (Genistein: 25 μg/mL, Quercetin: 10μg/mL, Polydatin: 100 μg/mL, ZnPCA: 25 μg/mL, Ferulic acid: 10 μg/mL, TXA: 10 μg/mL) Scale bar = 60 μm (upper panel), 18.75 μm (lower panel).

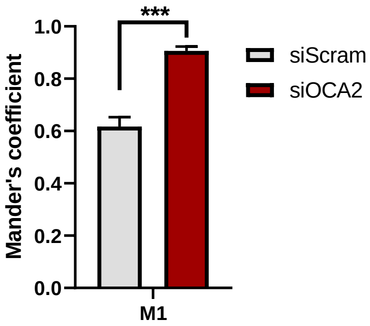

Supplementary Figure S6. Colocalization analysis between Lysotracker and TYRP1 following OCA2 KD in B16F10 cells. Calculation of Mander's co-localization coefficient of TYRP1 signal overlapping Lysotracker. The Mander's coefficient is 0.6 when siScram transfected and about 0.9 when siOCA2 transfected. The coefficient value increases significantly upon OCA2 KD. Error bars indicate the standard error of the mean. *** $p < 0.001$; (n = 557, 461 for each group; Student's t-test).

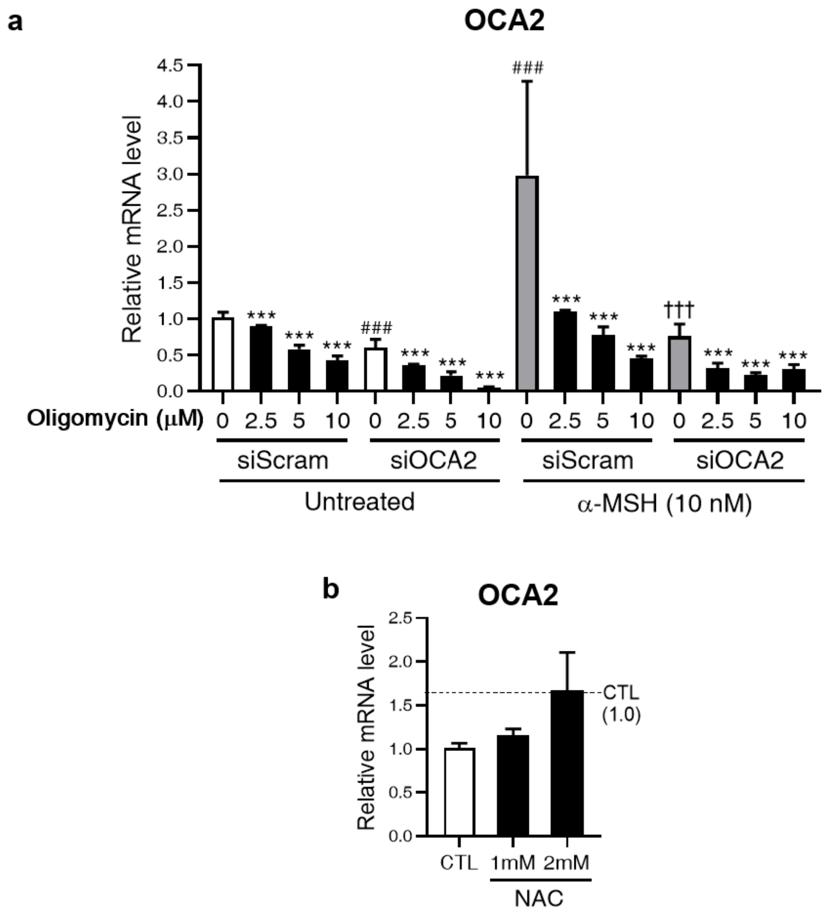

Supplementary Figure S7. Reduction of OCA2 expression by treating with oligomycin, mitochondrial ATPase inhibitor in B16F10 cells. **(a)** Decrease of OCA2 expression by treating with the oligomycin in a concentration-dependent manner. (n = 3, with duplicates for each group) **(b)** Treatment with N-acetylcysteine (NAC), the artificial antioxidant in B16F10 cells. It did not affect OCA2 mRNA levels. (n = 3, with duplicates for each group). Error bars indicate the standard error of the mean. Statistical significance was assessed using a student's *t*-test. Comparisons between groups are as follows: *** $p < 0.001$. Comparisons between group 1 and the other groups are as follows: ### $p < 0.001$. Comparisons between group 3 and group 4 are indicated as follows: ††† $p < 0.001$.

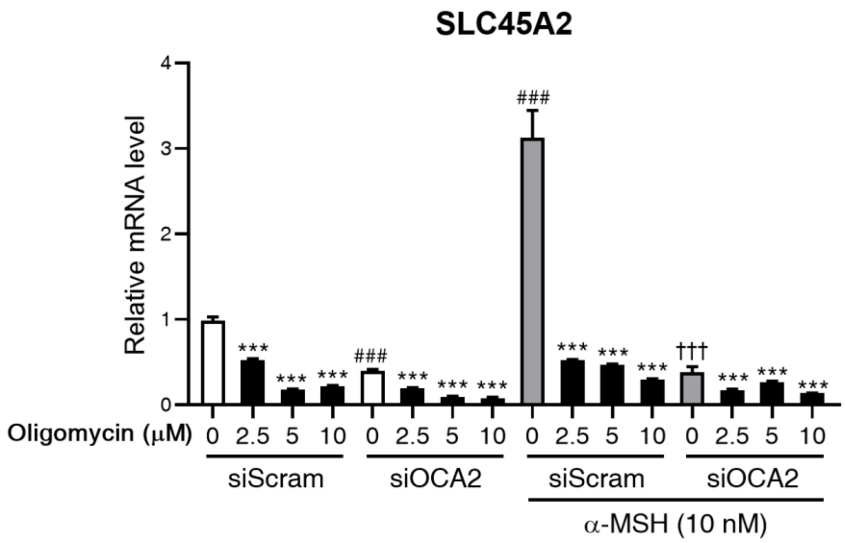

Supplementary Figure S8. Reduction of SLC45A2 mRNA level upon treatment with oligomycin in B16F10 cells. The relative mRNA expression decreased in a dose-dependent manner with increasing oligomycin concentration. SLC45A2 mRNA level was reduced upon treatment with oligomycin in B16F10 cells. The relative mRNA expression decreased in a dose-dependent manner with increasing oligomycin concentrations. (n = 3, with duplicates for each group). Error bars indicate the standard error of the mean. Statistical significance was assessed using a student's *t*-test. Comparisons between groups are as follows: *** p < 0.001. Comparisons between group 1 and the other groups are as follows: ### p < 0.001. Comparisons between group 3 and group 4 are indicated as follows: ††† p < 0.001.

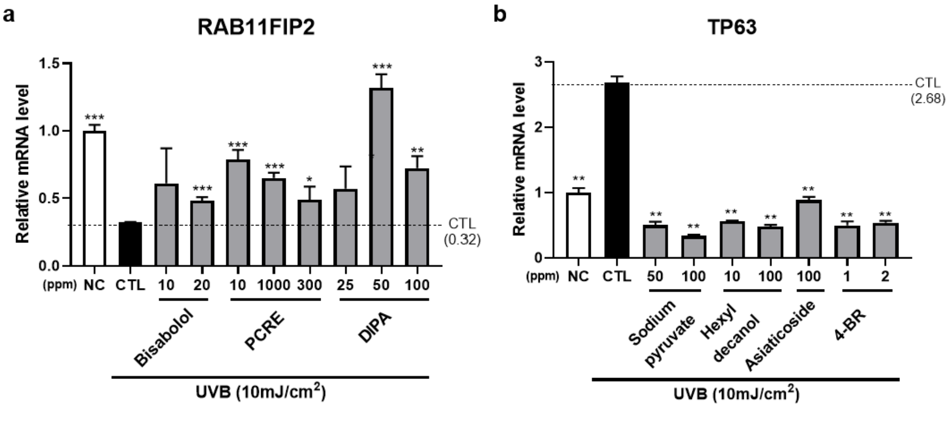

Supplementary Figure S9. Identification of expression regulatory substances for RAB11FIP2 and TP63 **(a)** Discovery of ingredients to restore the decreased expression of RAB11FIP2 upon UVB irradiation in B16F10 cells. Upon UVB (10 mJ/cm^2) irradiation, the expression of RAB11FIP2 is reduced. The expression was recovered upon treatment with bisabolol, *Polygonum Cuspidatum* Root Extract (PCRE) and diisopropyl adipate (DIPA). (n = 4 for each group). **(b)** Discovery of materials to suppress increased expression of TP63 upon UVB irradiation in HaCaT cells. Upon UVB (10 mJ/cm^2) irradiation, the expression of TP63 is increased. The expression level was inhibited by treating with sodium pyruvate, hexyl decanol, asiaticoside, and 4-butylresorcinol (4-BR). (n = 4 for each group). Error bars indicate the standard error of the mean. * $p < 0.05$, ** $p < 0.01$, *** $p < 0.001$; Student's *t-test*.

Supplementary methods

Cell culture

Human epidermal melanocytes, neonatal, light pigmented (HEMn-LP) were purchased from ThermoFisher Scientific Inc. (Waltham, MA, USA). The HEMn-LP cells were maintained in Cascade Biologics™ medium 254 supplemented with Human Melanocyte Growth Supplement (HMGS) (Gibco, S0025). Immortalized human keratinocytes (HaCaT) were purchased from Addexbio (San Diego, USA). The HaCaT cells were cultured in calcium free DMEM complete media with the addition of 0.01 mM Calcium chloride solution (sigma, 21115).They were incubated at 37°C in an atmosphere of 5% CO_2.

RT-qPCR

Supplementary Table S1

cells	Target gene	assay ID
B16F10	OCA2	Mm00498969_m1
	SLC45A2	Mm00499728_m1
	RAB11FIP2	Mm01261268_m1
	GAPDH (housekeeping)	Mm99999915_g1
HEMnLP	OCA2	Hs00609330_m1
	GAPDH (housekeeping)	(Cat# 4333764F)
HaCaT	TP63	Hs00978340_m1
	GAPDH (housekeeping)	(Cat# 4333764F)
