# Supplementary material for: Modulating OCA2 Expression as a Promising Approach to Enhance Skin Brightness and Reduce Dark Spots
Source: Biomolecules. 2024 Oct 11;14(10):1284. doi: 10.3390/biom14101284 (PMC11506640; doi:10.3390/biom14101284)
Supplement: Supplementary file 1 [file biomolecules-14-01284-s001.zip › Uncropped blot images_240829.pdf]

Supplementary Figure 1.

Supplementary Figure 1.

#1

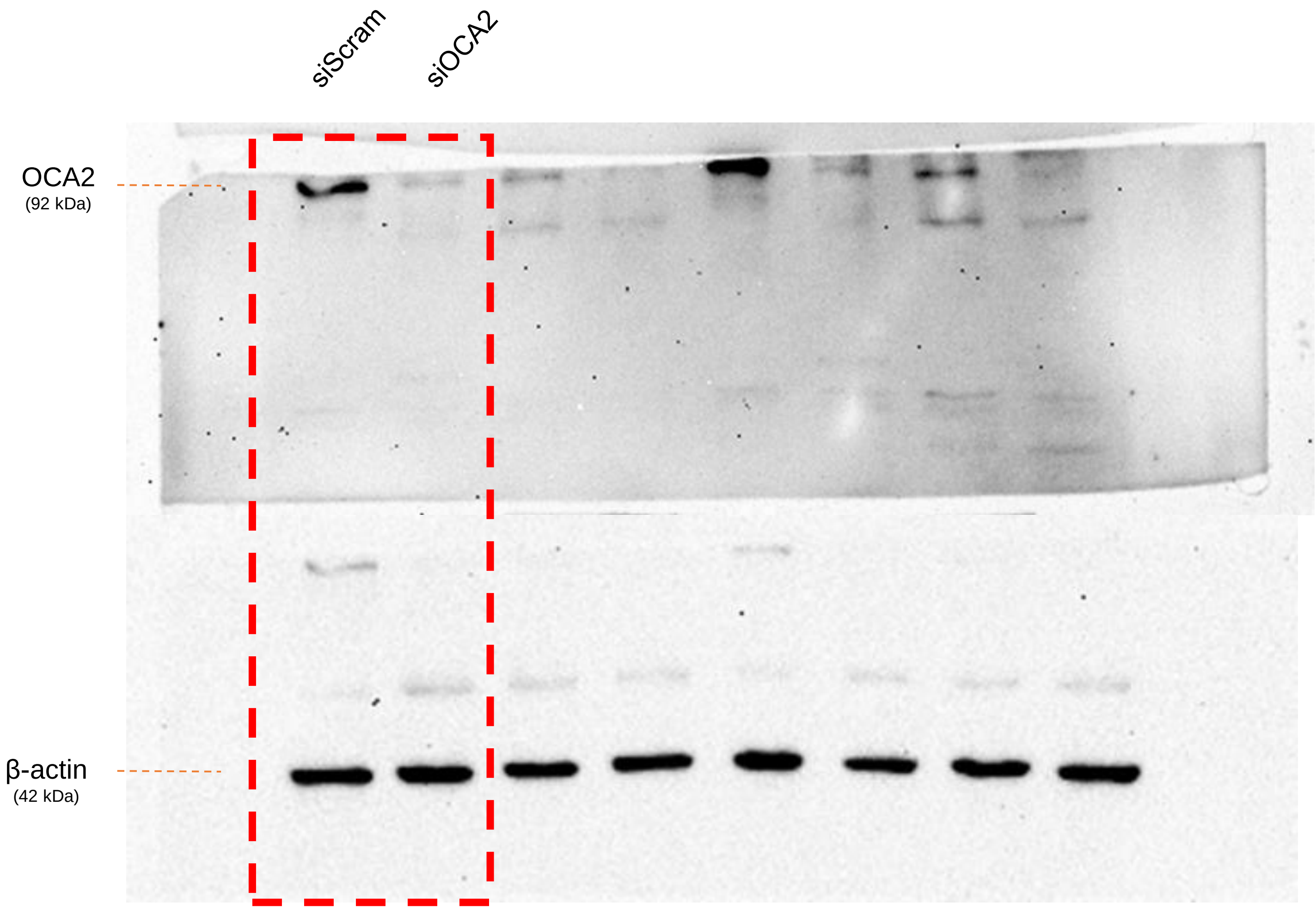

Supplementary Figure 1.

#2

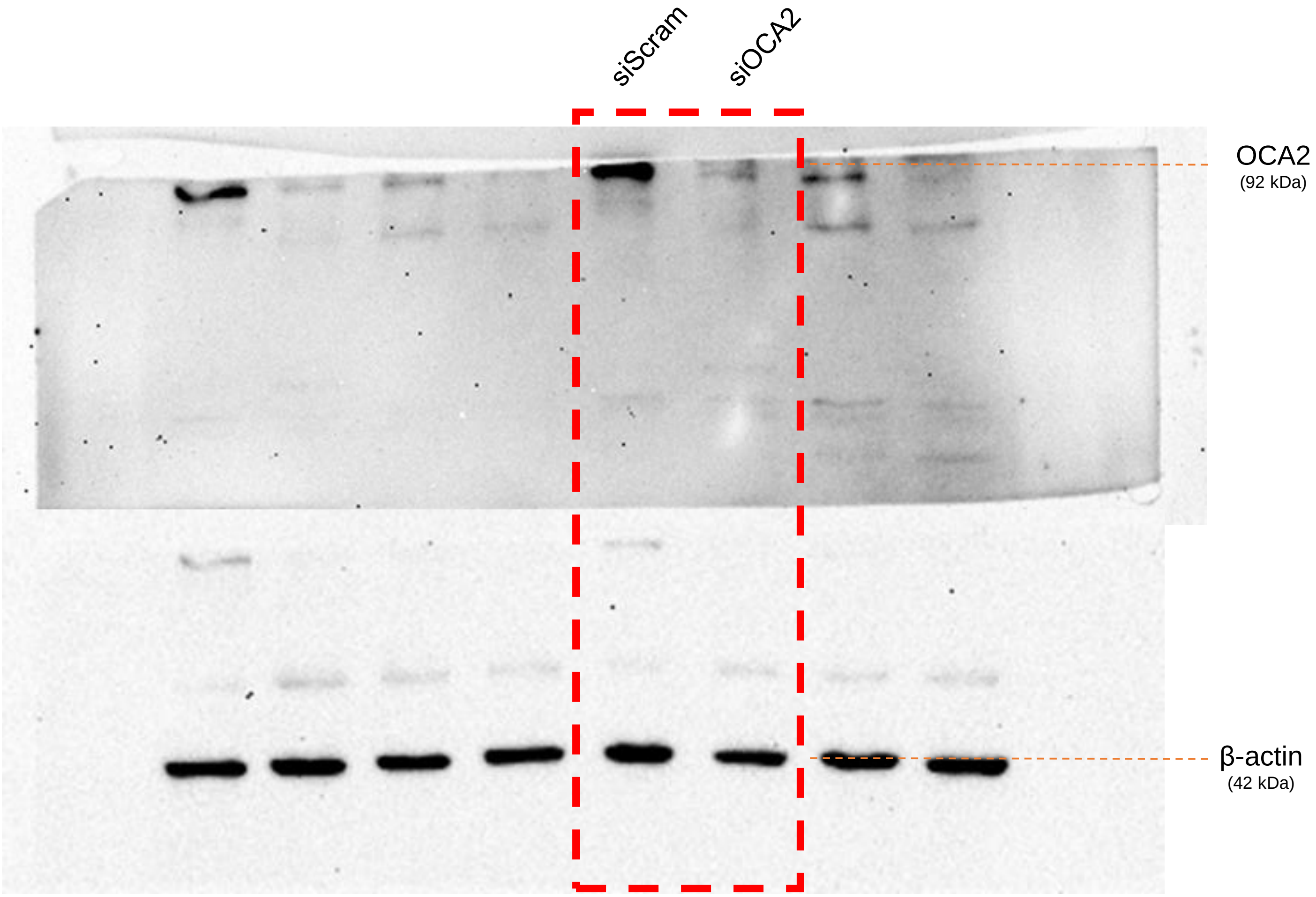

Supplementary Figure 1.

#3

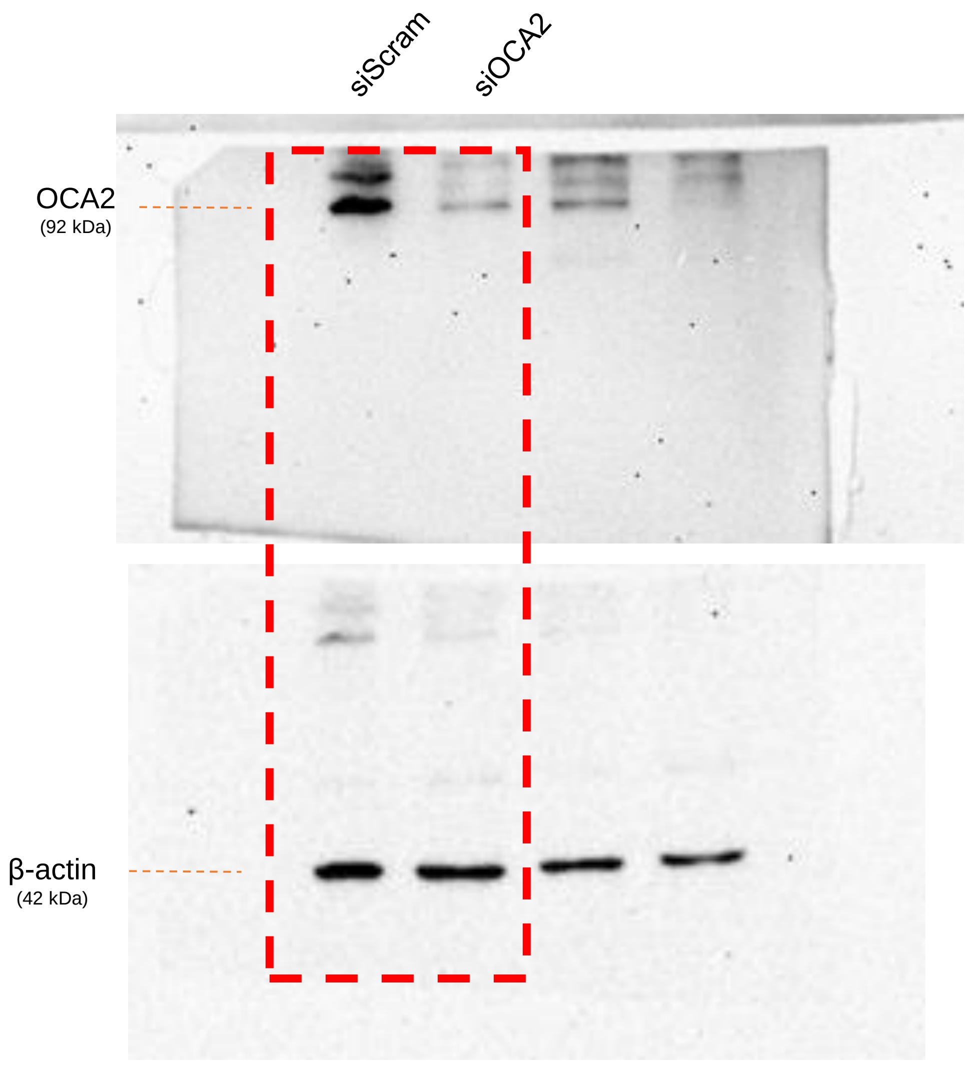

Figure 3. a.

Figure 3. a.

#1

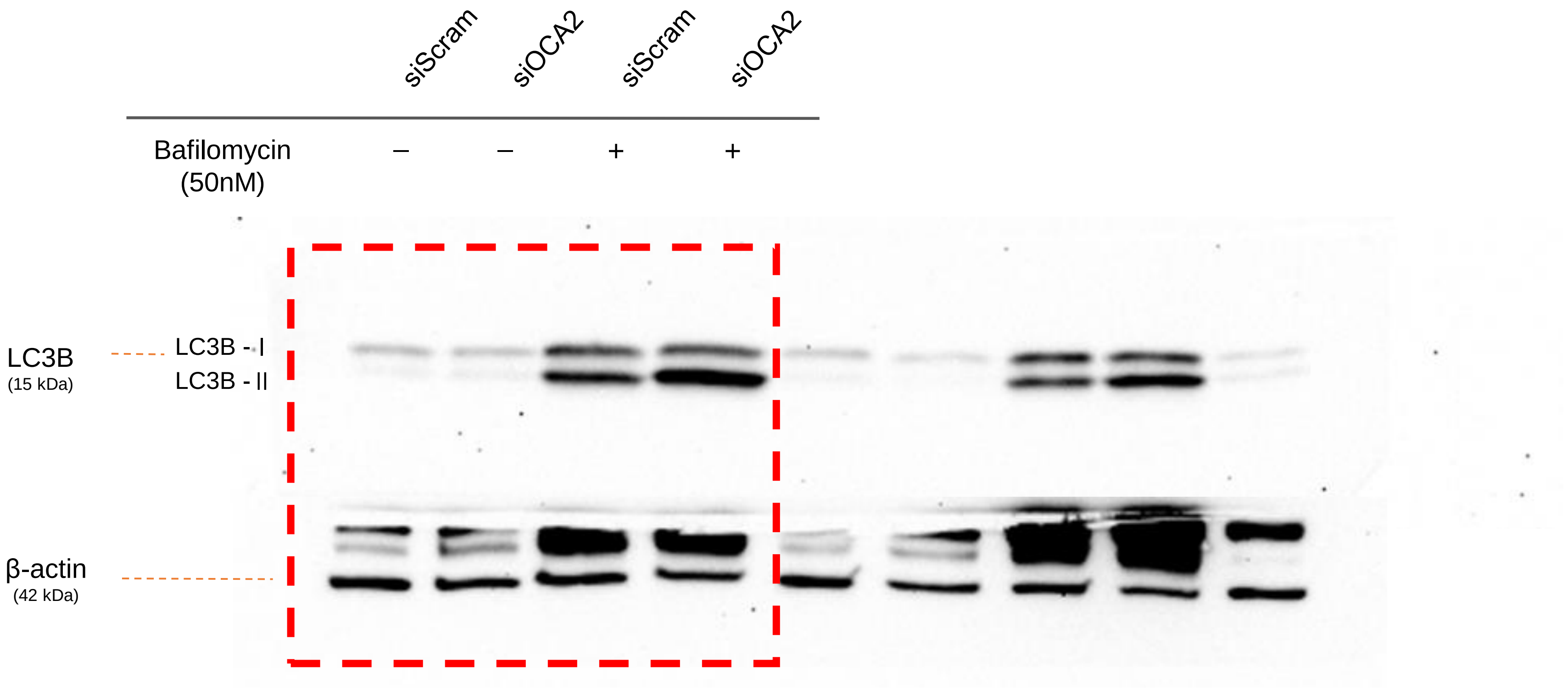
siScram
siOCA2
siScram
siOCA2
Bafilomycin (50nM)
–
–
+
+
LC3B
(15 kDa)
LC3B - I
LC3B - II
β-actin
(42 kDa)

Figure 3. a.

#2

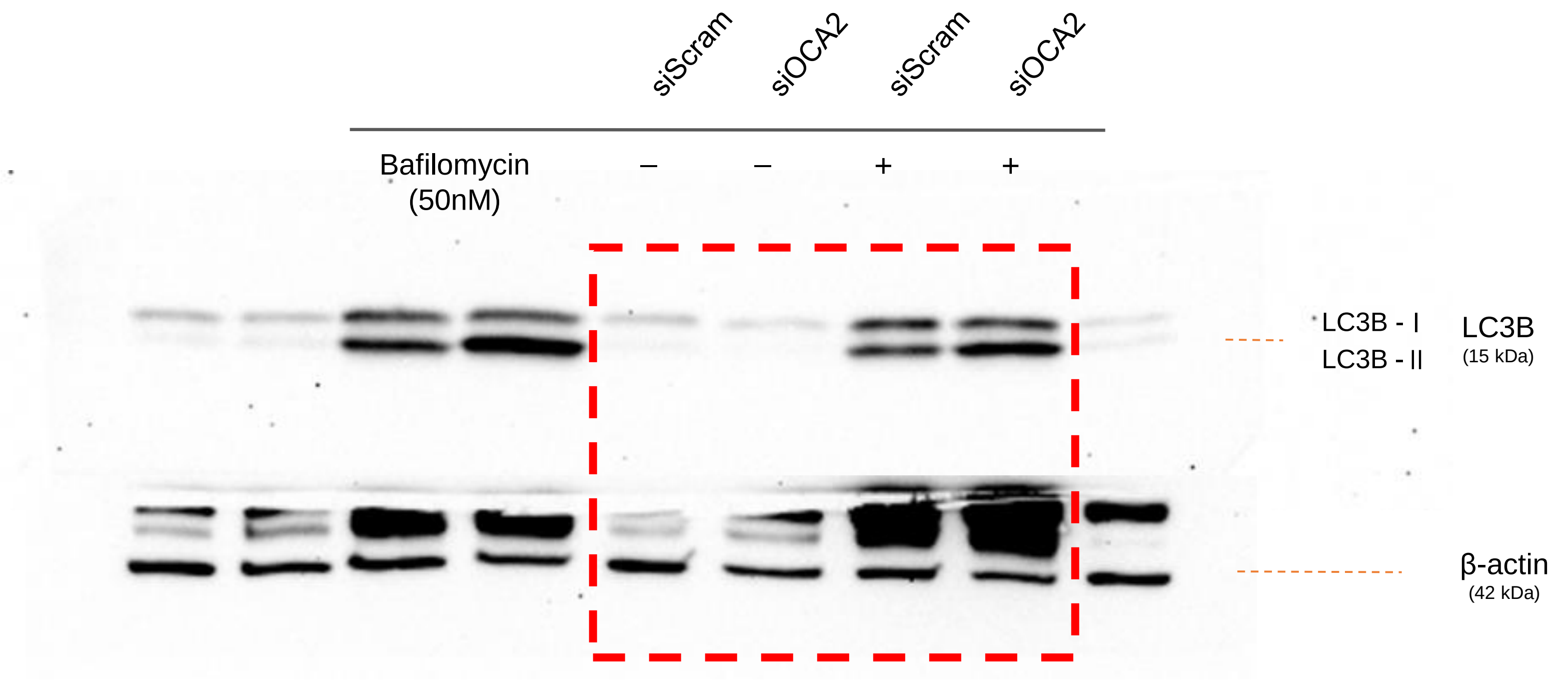
siScram
siOCA2
siScram
siOCA2
Bafilomycin (50nM)
–
–
+
+
LC3B - I
LC3B - II
LC3B
(15 kDa)
β-actin
(42 kDa)

Figure 3. a.

#3

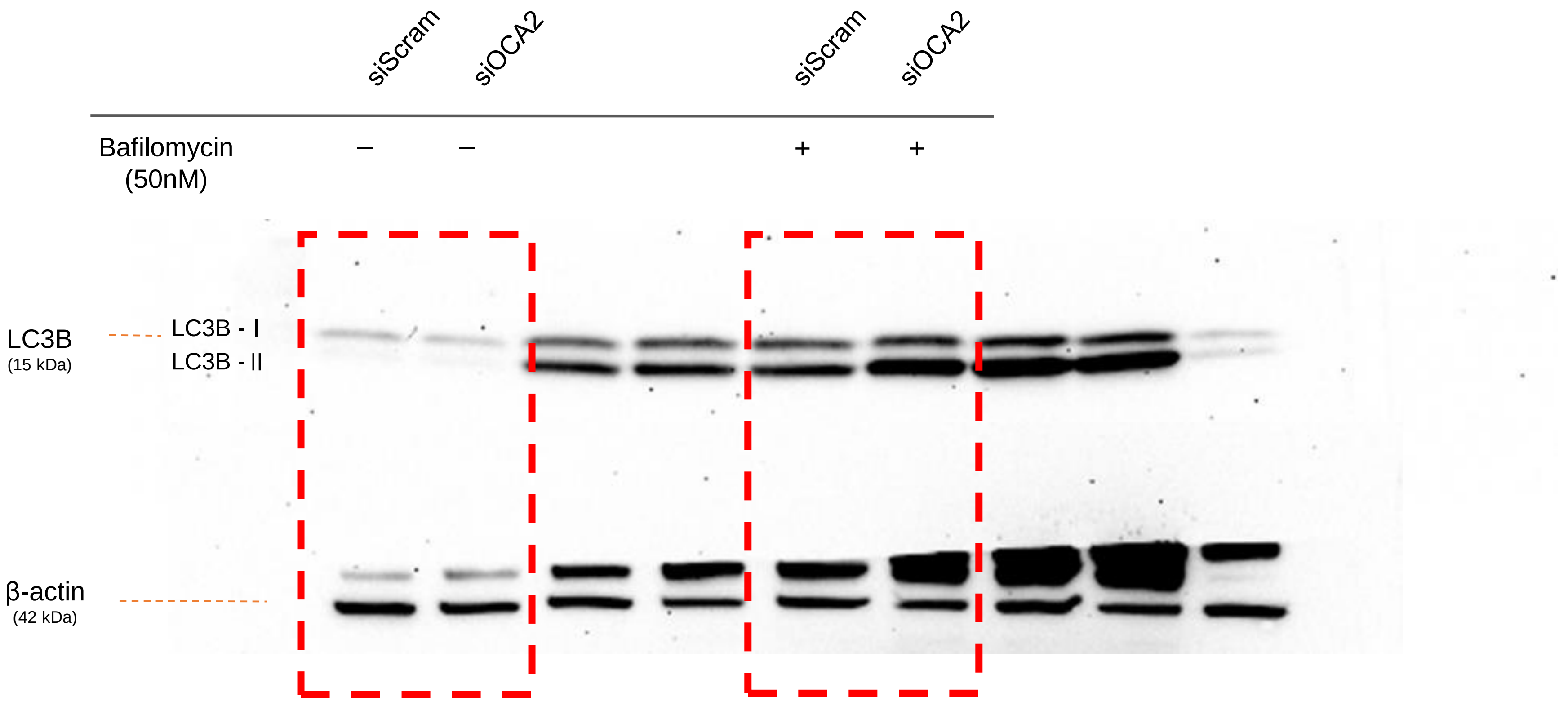

siScram
siOCA2
siScram
siOCA2
Bafilomycin (50nM)
–
–
+
+
LC3B
(15 kDa)
LC3B - I
LC3B - II
β-actin
(42 kDa)

Figure 3. a.

#4

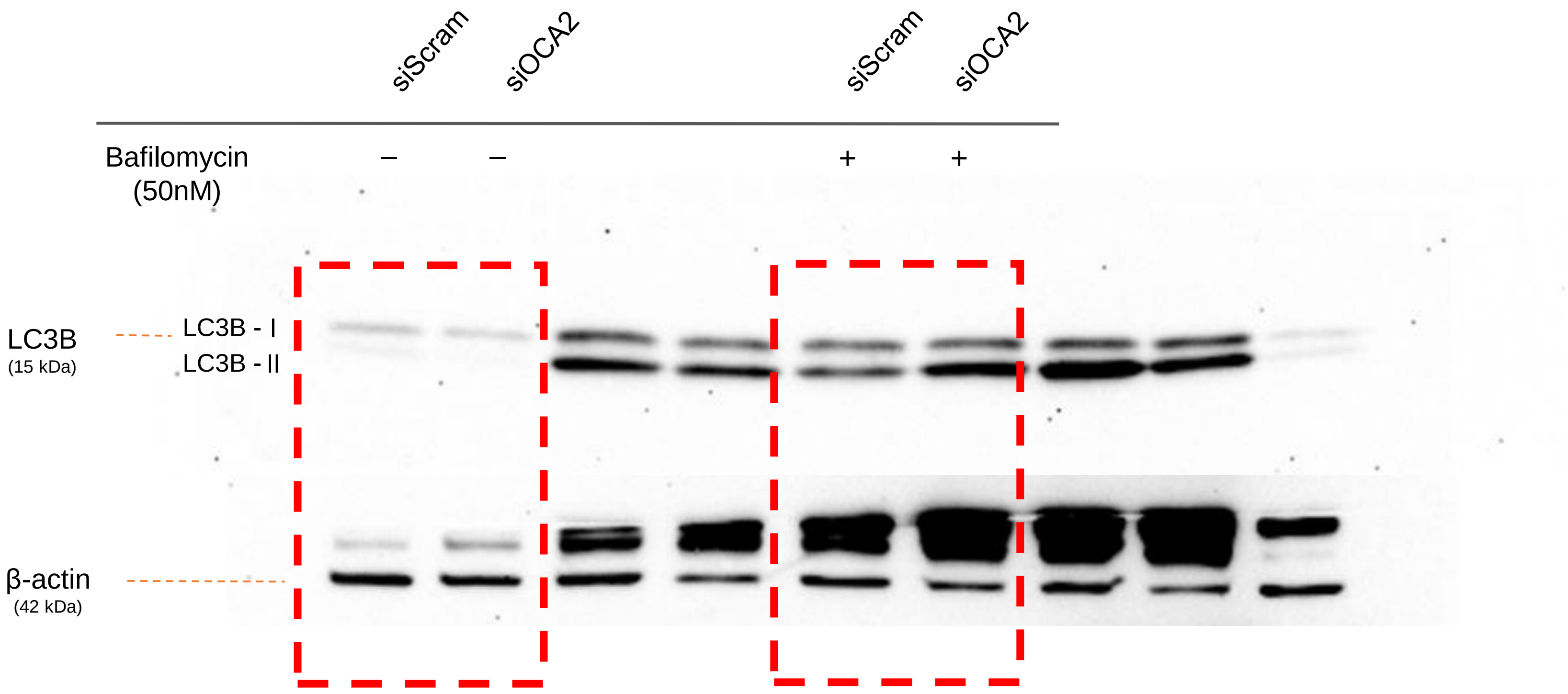

siScram
siOCA2
siScram
siOCA2
Bafilomycin (50nM)
–
–
+
+
LC3B
(15 kDa)
LC3B - I
LC3B - II
β-actin
(42 kDa)
